# Lobular Breast Cancer: A Review

**DOI:** 10.3389/fonc.2020.591399

**Published:** 2021-01-15

**Authors:** Natalie Wilson, Alastair Ironside, Anna Diana, Olga Oikonomidou

**Affiliations:** ^1^ Cancer Research UK, Edinburgh Centre, MRC Institute Genetics and Molecular Medicine, University of Edinburgh, Edinburgh, United Kingdom; ^2^ Edinburgh Cancer Centre, Western General Hospital, Department of Pathology, NHS Lothian, Edinburgh, United Kingdom; ^3^ Division of Medical Oncology, Department of Precision Medicine, School of Medicine, “Luigi Vanvitelli” University of Campania, Naples, Italy

**Keywords:** treatment, imaging, diagnosis, molecular features, lobular breast cancer

## Abstract

Invasive lobular carcinoma accounts for 5%–15% of all invasive breast cancers, with a marked increase in incidence rates over the past two decades. Distinctive biological hallmarks of invasive lobular carcinoma include the loss of cell adhesion molecule E-cadherin leading to cells with a discohesive morphology, proliferating into single-file strands and estrogen receptor positivity. These key molecular features can make diagnosis difficult, as invasive lobular carcinoma is challenging to detect both physically and with current standard imaging. Treatment of invasive lobular carcinoma strongly favors endocrine therapy due to low chemosensitivity and lower rates of pathological response as a result. This review will summarize the distinct biological and molecular features of invasive lobular carcinoma, focusing on the diagnostic challenges faced and the subsequent surgical and medical management strategies. Prospective therapeutic options will also be explored, highlighting how furthering our understanding of the unique biology of lobular breast carcinoma is essential in guiding and informing the treatment of patients in the future.

## Introduction

Invasive lobular carcinoma (ILC) accounts for 5%–15% of all invasive breast cancers (BCs) and is the second most common type of BC behind invasive ductal carcinoma (IDC) of no special type (1). Over the last two decades, there has been a marked increase in the incidence of ILC, mainly among the post-menopausal population. This is likely the result of improved diagnostic techniques and the use of hormone replacement therapy (2). ILC has a distinct biological profile and thus presents unique challenges with regard to systemic treatment and management of the disease. Hallmark features of ILC include; the loss of cell-cell adhesion molecule E-cadherin, resulting in small, discohesive cells proliferating in single-file strands, positivity for both the estrogen (ER) and progesterone receptor (PR), and human epidermal growth factor receptor 2 (HER-2) negativity (3). These key pathological features alongside the diffuse growth pattern of ILC make establishing a diagnosis particularly challenging. ILC is difficult to detect both upon physical examination and with standard imaging techniques. However magnetic resonance imaging (MRI) has a reported greater sensitivity in the detection and characterization of ILC than “gold standard” mammography. Systemic therapy is an integral part of the multidisciplinary approach to treating BC and this often involves the use of chemotherapy. However, due to the unique molecular biology of ILC, treatment response to chemotherapy is often predictably poor, resulting in lower rates of complete pathological response (pCR) thus leading to an increase in mastectomy rates in these patients (4). On the other hand, studies have shown that ILC responds well to endocrine therapy (ET), making it the optimal choice in the treatment of ILC (3, 5). The use of letrozole seems to provide greater overall survival (OS) benefit compared with tamoxifen, suggesting an increased incidence of endocrine resistance in ILC patients treated with tamoxifen (6). A deeper understanding of the unique molecular profile and alterations that define this BC subtype will lead the way in improving diagnosis, management, and treatment outcomes for patients with ILC. This review provides an up-to-date summary of the current understanding of ILC by discussing diagnostic challenges, surgical and medical management strategies, and prospective directions in the treatment of ILC.

## Incidence

ILC accounts for 5%–15% of all reported cases of BC (1, 7). On average, patients are 3 years older at diagnosis in comparison to IDC and are generally diagnosed at a more advanced stage of disease. Thus, tumors are often larger and show a greater degree of lymph node involvement at clinical presentation (7). The incidence of ILC has also increased over the past two decades, particularly in women over the age of 50 and is likely a result of diagnostic advances. It has also been correlated with the use of hormone replacement therapies, particularly those containing progesterone (8, 9). Several studies have suggested that the use of combined hormonal replacement therapy is related to a higher relative risk of BC (10–12). Since ILC is strongly ER-positive, it is unsurprising that prolonged and increased exposure to hormones represents a risk factor. Traditional hormone-related risk factors, including earlier menarche, later menopause, low parity, and late age at first birth, are all associated with increased incidence of ILC.

It has also been suggested that some lifestyle factors like alcohol consumption may play a role in ILC and IDC incidence. Among the first studies or even perhaps the first one that reported **alcohol** intake was positively related to ductal and **lobular tumors** was the study by Van’t Veer et al. (13). They showed that consuming more than 30 g of alcohol per day may enhance the BC risk in premenopausal women and that an early start to drinking alcohol may increase the relative risk for BC even beyond menopause (13). A great number of studies followed investigating the risk of alcohol intake and BC.

ILC is more frequent in the Western world while its incidence is much lower in the Middle East, Africa and Asia, accounting for only around 5% of BC cases in these regions. This is likely due to genetic factors (7). Hereditary ILC is rare but cases have been reported to occur as a secondary tumor in patients or families with hereditary diffuse gastric cancer syndrome who harbor a germline mutation of the *CDH1* gene (14). The risk factors of ILC are summarized in **Table 1**.

**Table 1:** A summary of the primary risk factors associated with the development of invasive lobular breast cancer.

Risk factors associated with Invasive Lobular Breast Cancer
Alcohol consumption Use of combined hormone replacement therapy Early menarche (defined as menarche before the age of 12 years) Late-onset menopause (defined as menopause after the age of 55 years) Nullparity/low parity (defined by WHO as less than 5 pregnancies with gestation periods of ≥20 weeks) Late age at birth (>30 years) Family history, e.g., hereditary diffuse gastric cancer syndrome Genetics, e.g., *CDH1* mutations

## Morphological Features and Immunophenotype of ILC

**Figure 1:**
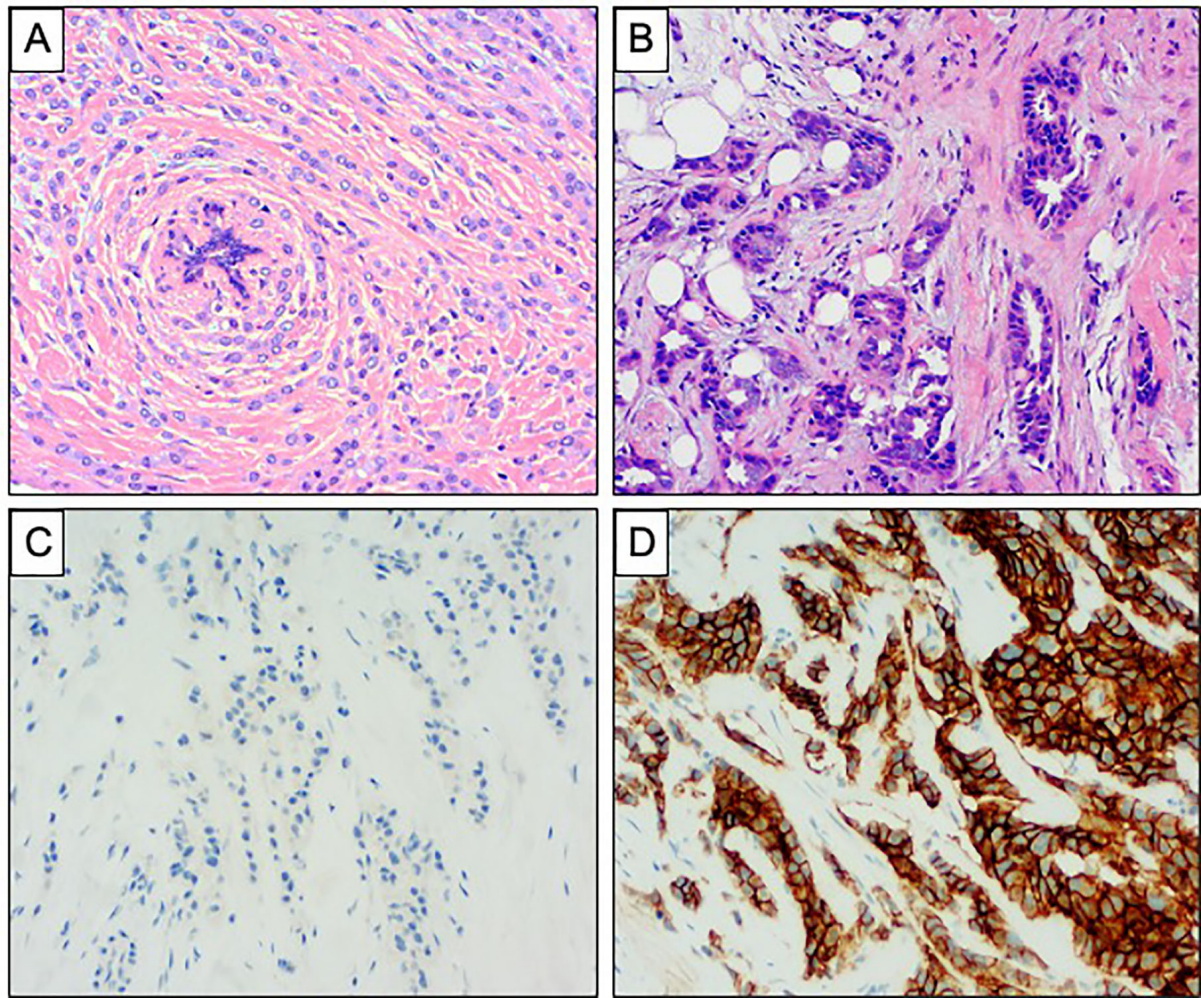
Comparison of invasive lobular carcinoma and invasive ductal carcinoma of no special type. **(A, B)** Haematoxylin and Eosin stained sections demonstrating the morphology of ILC and IDC of no special type. **(A)** ILC showing diffuse infiltration of the stroma with a single file pattern, surrounding a normal breast duct in a concentric manner. **(B)** IDC showing more cohesive tumor cells forming tubules with destructive infiltration of the mammary stroma. **(C, D)** Comparison of E Cadherin expression in ILC and IDC of no special type. **(C)** Complete absence of staining is seen in ILC. **(D)** Strong and diffuse membranous expression is seen in IDC.

**Figure 2:**
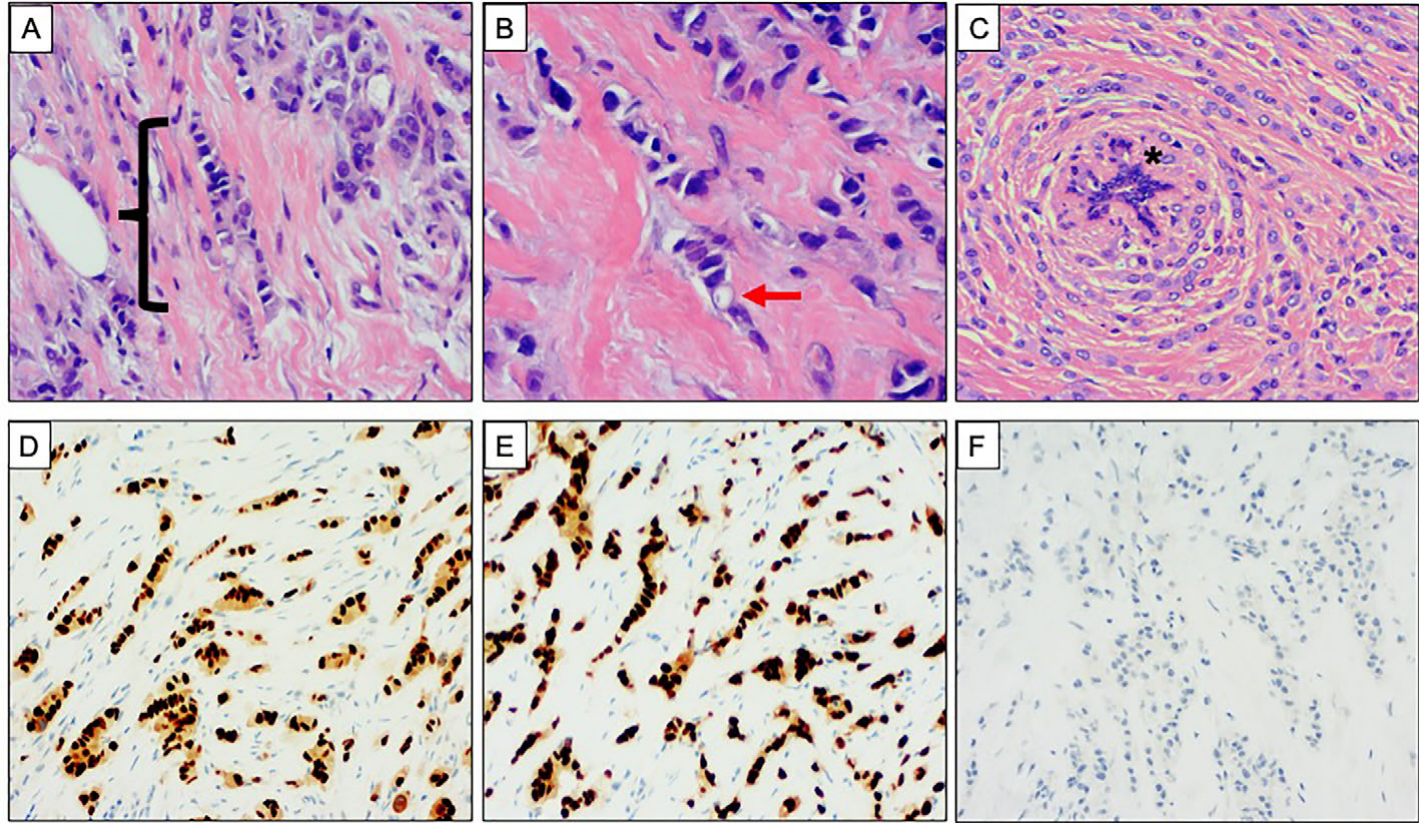
Classical morphology and immunophenotype of invasive lobular carcinoma. **(A**–**C)** Haematoxylin and Eosin stained sections demonstrating the classical morphology of invasive lobular carcinoma (ILC). **(A)** Single file pattern of invasion, highlighted with bracket ({}. **(B)** High power view showing discohesive tumor cells. An intracytoplasmic vacuole is highlighted with the red arrow. **(C)** Concentric pattern of infiltration around a normal breast duct, highlighted with asterisk (*). **(D**–**F)** Classical immunophenotype of ILC. Strong and diffuse nuclear expression of **(D)** oestrogen receptor and **(E)** progesterone receptor. **(F)** Absence of membranous E Cadherin expression.

**Figure 3:**
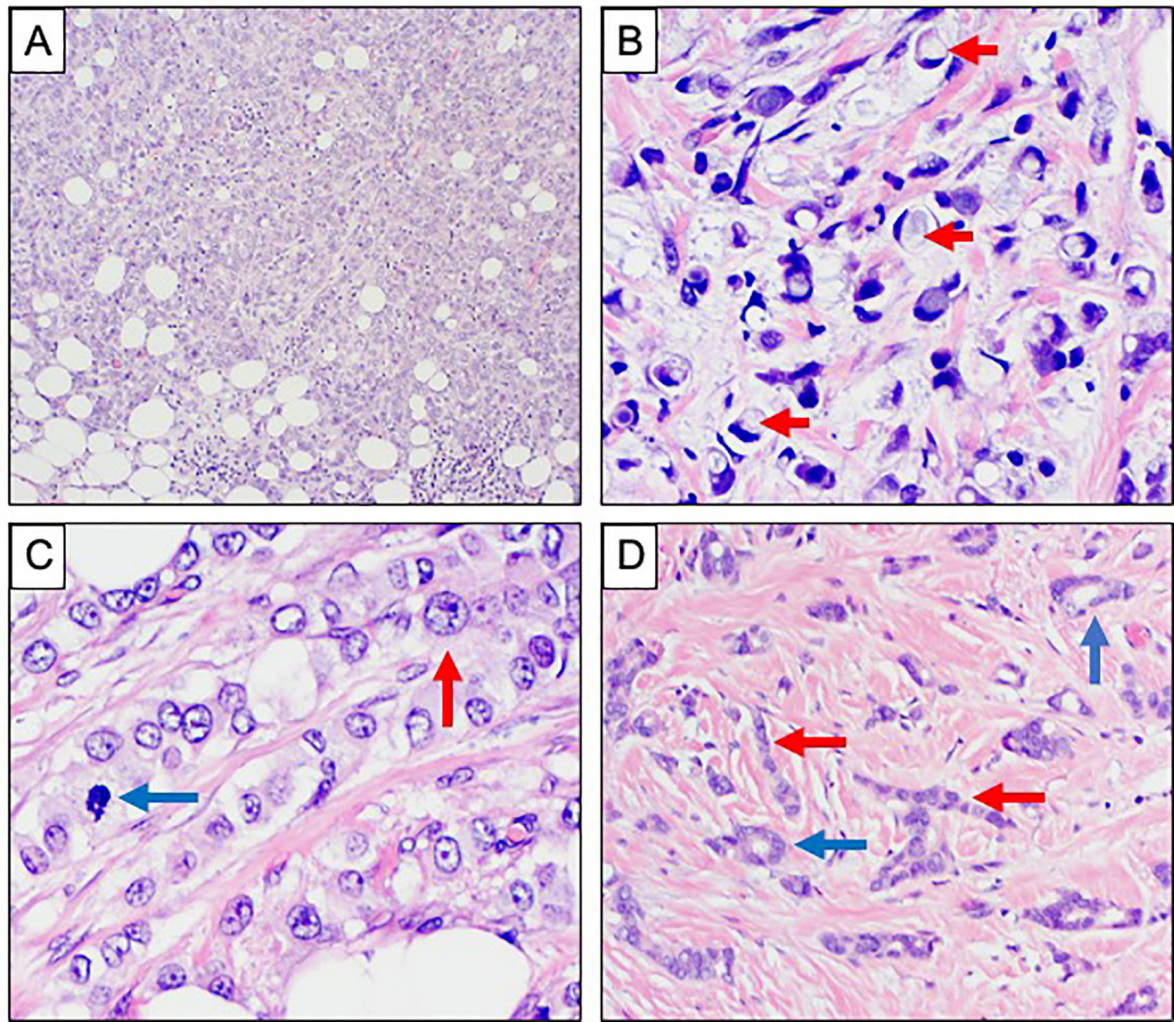
Morphological variants of invasive lobular carcinoma. **(A**–**D)** Haematoxylin and Eosin stained sections showing morphological variants of ILC. **(A)** Solid growth pattern. **(B)** Signet ring morphology with prominent intracytoplasmic mucin vacuoles, highlighted with red arrows. **(C)** Pleomorphic ILC showing more prominent nuclear atypia with conspicuous nucleoli (red arrow) and mitotic activity (blue arrow). **(D)** Tubulolobular carcinoma showing tubule formation (blue arrows) alongside the classical single file pattern of invasion (red arrows).

ILC often displays favorable characteristics that are associated with a good prognosis, typically being strongly ER positive and of a lower histological grade. As many as 95% of ILC cases express the ER and up to 70% of cases express the PR (15–18). By comparison only 60%–70% of IDC express both ER and PR. **Figure 1** shows the differences in morphological features between ILC and IDC. ILC can demonstrate a number of histological variants, these include classic, solid, alveolar, mixed, tubulo-lobular, and pleomorphic lobular carcinoma (1). Small, round, discohesive cells that are often described as having signet-ring cell morphology define the classic subtype. The distinct growth pattern features cells growing in linear strands through the stroma, with little disturbance to the surrounding breast tissue architecture (**Figure 2**). This arrangement of cells also forms in concentric patterns around structures such as ducts and lobular units (17). In addition, classical ILC is associated with low to moderate nuclear pleomorphism and a low mitotic index (1). The low proliferative index demonstrated by the majority of ILC may contribute to the lack of chemo-responsiveness. Histological variants of ILC can be identified either by their growth patterns or their cytology. The histology of the solid variant of ILC differs from classic type by forming large solid sheets of neoplastic cells, that can potentially be mistaken for other tumors such as lymphoma. This variant is commonly associated with high nuclear pleomorphism and a higher mitotic index (16). The alveolar variant is cytologically similar to classic ILC but cells tend to grow in groups of at least 20 cells, forming globular aggregates (17). The presence of tubular structures in association with the distinct filing growth pattern defines the tubulo-lobular variant of ILC (16). Pleomorphic lobular carcinoma was first described in 1982 by Dixon et al. and is characterized by a greater degree of nuclear atypia and pleomorphism and a higher mitotic index, conferring a more aggressive phenotype than classic ILC (18). The pleomorphic variant does retain the classic ILC pattern of single cell files, even if the architecture of the lesion is often mixed (**Figure 3**). This aggressive phenotype is often associated with a poorer prognosis when compared to other ILC variants (19). Weidner et al. (20) reported that patients with pleomorphic ILC were four times more likely to experience recurrence than patients affected by classic variant and Orvieto et al. (21) conducted an analysis of 530 cases of ILC (57% classic, 19% alveolar, 11% solid, and 13% displayed pleomorphic or apocrine features). In this study, classic histology was associated with a lower risk of lymph node metastasis and a lower tumor grade compared to non-classic histology, which showed an increased number of distant metastases and a significant reduction in disease-free survival (DFS) and OS. Talman et al. (22) evaluated 860 tumors of patients with classic or non-classic ILC, regarding subtype and grade in relation to prognosis. The results showed that most cases of classic ILC were grade II tumors, with a small portion being grade I but prognosis remained the same despite this. There was a higher frequency of grade III tumors among solid and pleomorphic subtypes and this was associated with a significantly worse survival outcomes compared to grade II tumors. A study by Iorfida et al. (23) featuring 981 ILC patients, reported a longer DFS and BC-specific survival (BCSS) in those with classic ILC rather than patients with solid and mixed variants. Despite the overall good prognosis associated with classic ILC, tubulo-lobular variants may exhibit the best prognosis of all ILC subtypes. Noteworthy, du Toit et al. (24) investigated 171 cases of ILC over a period of 11 years reporting a 12-year survival rate of 100% for tubulo-lobular subtype. This excellent prognosis is reflected in the fact that tubulo-lobular tumors are likely to be of low histological grade and are often node negative. In contrast, patients with pleomorphic ILC generally have a poorer prognosis, presenting at an advanced stage of disease with larger tumors and lymphovascular invasion (25). Biomarkers associated with poor clinical outcomes are rarely displayed in ILC; however, pleomorphic ILC is an exception to this rule, exhibiting a lack of ER and PR expression, HER2 amplification, p53 expression and higher proliferation rates (16).

## Molecular Features of ILC

The characteristic feature of ILC is the loss or lack of E-cadherin expression (26). E-cadherin is an essential molecule in mediating cell-cell adhesion in order to maintain cell viability; dysregulation results in the distinctive discohesive growth pattern observed in ILC. E-cadherin is able to from adherens junctions between cells through association with α-, β-, γ-, and p120 catenins, together they work to maintain cellular cohesion. Approximately, 90% of ILCs lack E-cadherin expression, a feature that is important in the diagnosis and classification of ILC, particularly when differentiating from IDC. In addition to the loss of E-cadherin, α-, β-, γ-catenins are also lost in ILC but p120-catenin is up-regulated and localized to the cytoplasm, acting as another biomarker of ILC (16). The hallmark loss of E-cadherin is driven by alterations to the CDH1 gene, located on chromosome 16q22, which codes for E-cadherin. Despite the strong association between CDH1 mutation or deletion and the hallmark loss of E-cadherin in ILC, mechanisms underlying this alteration are not well studied (27). The most likely explanation for the inactivation of CDH1 follows the “two-hit hypothesis” model where a first-event somatic mutation is followed by loss of heterozygosity or gene methylation. Mutations in CDH1 have been identified in other epithelial cancers, most notably in diffuse gastric cancer, which shares similar features with ILC (28). Namely, the appearance of neoplastic and signet ring cells in an infiltrative growth pattern (16). The International Gastric Cancer Linkage Consortium studied 11 CDH1 families and interestingly found that in addition to diffuse gastric cancer, female gene carriers were also at a higher risk of developing ILC (14). Overall, this suggests CDH1 inactivation is a key event in the pathogenesis of ILC (29). CDH1 is not the only genomic alteration associated with ILC.

**Figure 4:**
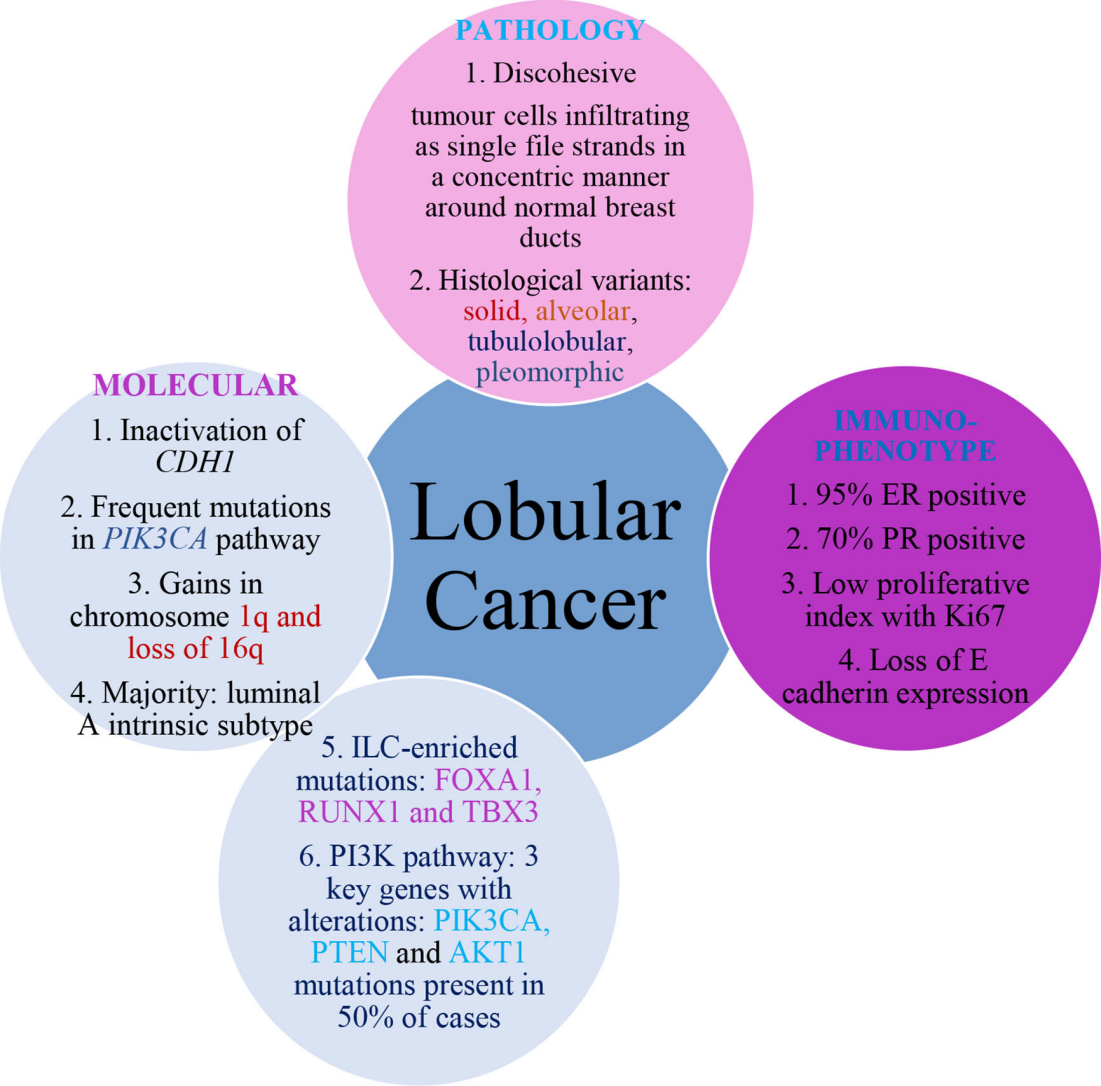
Molecular, pathology, and immunophenotype features of ILC.

The Cancer Genome Atlas (TCGA) study has also identified a number of ILC-enriched mutations including FOXA1, RUNX1, and TBX3 (5). FOXA1 expression is high in BC and mutations are present in approximately 7% of all ILC cases (30). FOXA1 is as a key transcription modulator of ER activity, therefore mutations can affect ER function as the loss of ER-binding blocks ER-mediated gene expression, altering the response of endocrine targeted therapies such as Tamoxifen (3). In a study conducted by Desmedt et al. (31), aimed to characterize the genome of 630 ILC tumors, CDH1 mutations occurred in 65% of tumors. However, the phosphatidylinositol 3-kinase (PI3K) pathway showed three key genes with alterations: phosphatidylinositol-4,5-bisphosphate 3-kinase catalytic subunit alpha (PIK3CA), phosphatase and tensin homolog (PTEN) and AKT1 mutations were present in 50% of cases. PIK3CA mutations were associated with low proliferation rates, as defined by Ki-67 and AKT1 tumors were related to a short-term risk of relapse. While loss or inactivation of PTEN can result in a more aggressive phenotype (**Figure 4**) (3).

ILC is overwhelmingly characterized by HER2 negativity, displaying a low rate of ERBB2 amplification. However, ERBB2 mutations or amplifications have been found in up to 8% of ILCs. These mutations are most associated with pleomorphic and solid histology and this is thought to account for the aggressive tumor phenotype (31, 32). Outside of mutations in CDH1 and members of the PI3K pathway, few other potential driver mutations in ILC have been identified. ILC harbors fewer chromosomal alterations than IDC, typically exhibiting a high frequency of gain of chromosome 1q and loss of 16q (33, 34). Gene expression profiling studies have categorized BC into four “intrinsic subtypes”—luminal A, luminal B, Her 2 enriched and basal like (35). ILC are frequently classified as luminal A, in keeping with the majority of tumors being low grade and ER positive. However, significant heterogeneity exists and ILC have the potential to be classified as any of the four main intrinsic subtypes (36). A better understanding of the molecular and genomic alterations that characterize ILC will facilitate a more personalized approach to treating BC.

## Diagnosis of Lobular Cancer

Diagnosis of ILC by physical exam can be challenging as patients often present with limited clinical signs and do not always have a palpable breast lump; signs may be frequently vague such as skin thickening or dimpling. Measuring the extent of ILC can be difficult as traditional screening methods such as mammography and ultrasound (US) have a low sensitivity for detecting ILC compared to other invasive breast tumors. This difficulty can be largely attributed to the diffuse infiltrative growth pattern of ILC (2).

### Mammography

Mammography is considered the “gold standard” imaging method in the early detection of BC (1) with a sensitivity typically ranging from 63% to 98%. This is achieved by producing high-resolution images, highlighting the contrast differences between healthy and malignant breast tissue (37). Detection of ILC using mammography is notoriously challenging due to the infiltrative tumor growth pattern which does not destroy the underlying anatomic structures or incite a desmoplastic stromal reaction. Due to these uncommon tumor characteristics, the sensitivity in detecting ILC is much lower, ranging between 57% and 81%. False positives are also not uncommon with reported rates ranging from 8% to 24% (2). A study by Krecke and Gisvold reported that the false-negative rate in the diagnosis of ILC is much higher than that for other invasive BCs (38). Furthermore 54% of mammograms that were deemed to show no evidence of malignancy were later found to be suggestive of a tumor. Upon reviewing these mammograms, 46% still concluded no visual evidence of malignancy. The inverse relationship between breast tissue density and mammographic sensitivity is well established. In the case of extremely dense breast tissue, mammographic detection can be as low as 30% (37). In a study by Berg et al. (39) mammographic sensitivity was found to be around 34% in cases of ILC and when adjusting for patients with dense breast tissue sensitivity decreased to just 11%. In addition to its distinct histological growth pattern, low opacity may also explain the challenges of clinically identifying ILC with mammography (40). A study by Hilleren et al. (41) reported that up to 50% of ILC have a lack of opacity which is less than or equal to normal breast tissue upon imaging. This lack of contrast highlights the challenge in delineating between malignant and normal breast tissue using conventional mammography in ILC (37). Invasive carcinomas are often associated with high density spiculated masses, due to the disruption of normal breast tissue architecture. This kind of mass can be easily detected by mammography. Reports suggest that ILC manifest as a poorly spiculated and ill-defined lesions, with a well-defined mass seen in less than 1% of cases (37). In addition, up to 35%, of ILCs are reportedly only visible on one view, this is most often the craniocaudal view (2). This may also contribute to estimations of tumor size and extent being less reliable (42). Microcalcifications are often considered common indicators of breast disease and are readily detected by mammography, however the likelihood of ILC producing calcifications is low (43). The presence of calcifications associated with ILC reportedly ranges from 1% to 28%. This is another hallmark feature of ILC that contributes to the inability of mammography to readily detect these tumors. Given all of the above it is unsurprising that the threshold for recommending additional imaging methods remains low (37).

### Ultrasound

US is another diagnostic breast imaging tool that is most commonly used in conjunction with mammography. US was originally used as a tool to differentiate between solid and cystic lesions and to guide biopsy procedures (44). However, with technological advances, US now has improved sensitivity to separate benign from malignant lesions and is used in the investigation of all palpable breast lumps (37). The relationship between breast density and mammography sensitivity is well documented however with the addition of US, there may be up to a 40% increase in the detection of asymptomatic cancers, which often include ILC (40). The reported sensitivity of US in the detection of ILC ranges from 68% to 98% (37, 45). The most common sonographic features of ILC are an irregular, hypoechoic mass with ill-defined margins and posterior shadowing, observed in up to 61% of cases (46). Well circumscribed masses are rarely seen in lobular tumors, manifesting in as little as 2% to 12% (37). When comparing the sensitivity of US to mammography, it would appear that the former is a more valuable imaging tool in the detection of ILC. Porter et al. (47) investigated the use of mammography and US in ILC, reviewing 361 cases diagnosed between 1995 and 2010. This study found that false-negatives occurred in 29.9% of cases when using mammography, while US had a reported sensitivity of 97.8% in detecting ILC associated abnormalities. Butler et al. (48) also evaluated the use of US in cases of ILC. A total of 208 cases were reviewed and of these 81 tumors were considered invisible on mammography or mammographically subtle. Results showed that 73.3% of cases that were considered mammographically invisible were identified by US. Similarly, 91.2% of tumors that were “mammographically subtle” were visualized using US. Thus, US is a helpful adjunct in the diagnostic pathway, particularly when faced with a suspicious physical exam and reportedly normal mammogram (37). The use of US has proved valuable in significantly improving the detection of ILC (44).

### Magnetic Resonance Imaging

**Figure 5:**
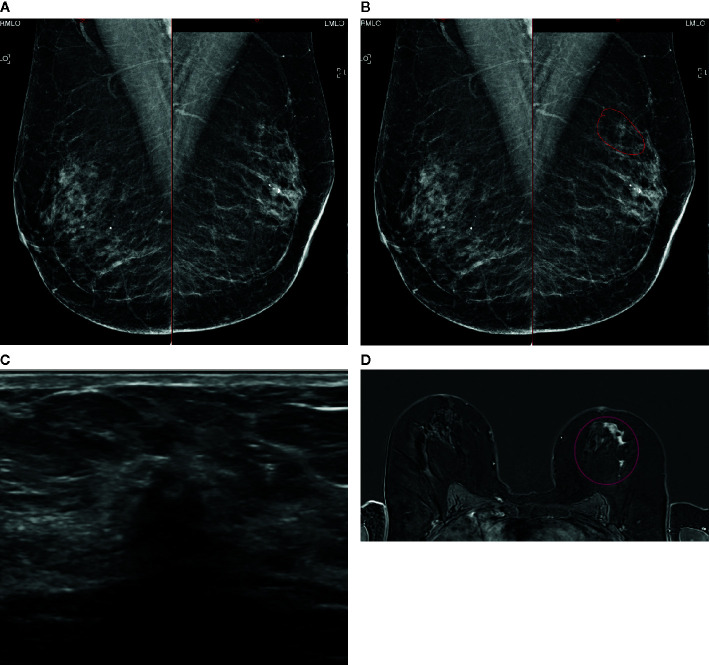
A case of extensive ILC in a 50-year-old female attending for 1^st^ breast screening. **(A)** Mammogram: Right and left MLO views. **(B)** Mammogram is reported as M3. There is a 5-mm area of concern on the left which is circled. The calcifications are considered benign. The patient does not feel a palpable mass. **(C)** An US of the left breast is performed and shows a small 8mm focus close to the nipple. **(D)** MRI breast follows and this shows an extensive area measuring approximately 50 mm × 50 mm (circled). Biopsies were performed and showed Gr2 ILC ER8 PgR8 HER negative. Patient had mastectomy and sentinel node biopsy (SNB) that showed 52-mm Gr2 ILC ER8 PgR8 HER2 negative SNB 0/3. **(A)** Mammogram: Right and left MLO views **(B)** Mammogram M3: possibly a 5-mm area of concern on the left (circled). Calcifications look benign. **(C)** Ultrasound: One small focus closer to nipple maybe 8mm. **(D)** MRI: Extensive central disease over 50 mm × 50 mm (circled).

MRI is mostly used in the screening of high-risk BCs, to evaluate and compare mammographic and US findings, assess chemotherapy response and to evaluate ipsilateral and contralateral breast tumors. MRI has a high overall sensitivity of 90% in the detection of BC and a sensitivity of 93% in detecting ILC (37). This high level of sensitivity is based on the increased levels of neovascularization in tumors as they constantly create new blood vessels in a bid to provide nutrients for further tumor growth. This results in a rapid uptake of gadolinium-based contrast, which can accumulate in the BC stroma (49). This high sensitivity also extends to the increased detection of multifocal, multi-centric and contralateral disease (50). MRI detects additional tumor foci and contralateral breast disease in between 16% and 58% of patients with ILC, not detected on initial mammogram (2). Rodenko et al. (51) compared MRI and mammographic imaging in the management of ILC in 20 patients, demonstrating an agreement of 85% between pathology and MRI on tumor size and location. Conversely, the disease extent shown by mammography correlated with pathology in 32% of cases. This suggests that MRI is significantly more accurate than mammography in assessing disease extent in patients with ILC. MRI could be particularly valuable in newly diagnosed ILC to accurately assess disease extent, which may aid in pre-operative planning (37). A study by Bedrosian et al. (52) of 267 BC patients concluded that ILC patients were twice as likely to have their treatment regimen changed as a result of MRI than patients of any other histological subtype (2). Despite its high level of sensitivity in the early detection of BC, a considerable limitation of MRI is the lack of specificity (53). A low specificity can result in the over-treatment of patients resulting in extensive surgery with no added clinical benefit (50). Despite this, the presumed improvement of surgical outcomes and overall DFS as a result of MRI has been controversial. A retrospective study by Mann et al. (54) assessed the impact of MRI on re-excision rates in ILC and concluded that patients who had an MRI prior to surgery had significantly lower rates of re-excision (9%) than those who did not undergo an MRI (27%). This study also reported that MRI assessment did not result in a higher number of mastectomies being performed. On the other hand, more recently, a meta-analysis of pre-operative MRI on surgical outcomes reported that MRI significantly increased the rate of mastectomy across all BC subtypes and found weak evidence to support the claim that MRI can reduce re-excision rates in patients with ILC (55). Therefore, it cannot be claimed with confidence that the use of MRI reduces the rate of recurrence or DFS in ILC (37). Overall, MRI is particularly advantageous over standard imaging methods due to its increased sensitivity in detecting ILC tumors and improving the detection of ipsilateral and contralateral tumors (**Figure 5**). In conclusion, despite its limitations, MRI provides additional diagnostic information that may be missed during standard imaging and should be used in combination with US and mammography in accurately assessing patients with ILC (37, 50).

## Management and Treatment of ILC

Treatment protocols for ILC are in line with those used in all other subtypes of BC. However, features specific to the ILC subtype can impact both surgical and therapeutic management (3). A typical BC treatment plan involves a multidisciplinary approach comprising surgery, radiotherapy and systemic therapies.

### Surgical Management of ILC

The decision to proceed with surgery is usually determined on the basis of TNM stage at presentation, regardless of histology. Cancers that are deemed operable will usually be managed surgically upfront while some cancers may require neo-adjuvant therapy (NAC) to reduce tumor burden and facilitate surgical intervention. In the majority of cases, breast-conserving surgery in the form of a wide local excision can be performed, removing the tumor from surrounding healthy tissue, with clear surgical margins while maintaining the natural shape of the breast. However, it has been reported that in up to 65% of ILC cases, a second surgery will be required (3). ILC is characterized by a higher incidence of diffuse and multifocal lesions that are hard to detect both on imaging and intra-operatively, often resulting in re-excision or mastectomy following original breast-conserving surgery (56). In addition, axillary lymph node status is a crucial factor in BC prognosis and influences surgical planning. Sentinel node biopsy (SNB) is the standard method of assessing the axilla (57). SNB is used in patients with ILC however the ability to find metastatic deposits is challenging due to the discohesive nature of the cells (5). Axillary lymph node metastasis is an important predictor of patient survival, as the number of metastases increases, patient survival decreases. However, despite being typically diagnosed at a more advanced stage, ILC is not thought to be associated with an increased risk of lymph node metastasis (58). In the case of nodal involvement, an axillary node clearance should be performed. This is essential for prognostic purposes and ensures the lowest rate of axillary recurrence (59). Some studies have reported that the risk of local recurrence is greater in ILC patients however the data on local recurrence rates is dependent upon the duration of follow-up and few studies have specifically focused on ILC (3). A study by Vo et al. (60) evaluated treatment outcomes for breast conservation therapy in those with ILC and IDC. The 5- and 10-year recurrence rates for the ILC group were 1% and 7% compared to 4% and 9% for the ductal group, respectively. This suggests that breast-conserving surgery leads to similar outcomes despite tumor histology. Positive surgical margins are one of the most important factors in determining the risk of local recurrence. Chagpar et al. (61) reported that patients with a ILC had a greater likelihood of positive margins following a lumpectomy. Patients with ILC had positive margins in 15.8% of cases, compared to 9.8% of cases with IDC. This is likely due to the challenges in accurately assessing disease extent in ILC. A more recent study exploring the success of re-excision of positive margins in women with ILC reported that while initial positive margins occurred in 37.6% of cases, clear margins were achieved in 74.2% of patients who underwent a re-excision lumpectomy (62). This suggests improvements in the surgical management of ILC, and that completion mastectomies may not be required in patients who have positive margins following their initial breast-conserving surgery. In further support of breast-conserving surgery for ILC, Fodor et al. (63) concluded that BCSS was not affected by surgical treatment (breast-conserving surgery or mastectomy) in patients with ILC, spanning a 15-year period. Overall, this suggests that breast-conserving surgery can be safely implemented in the surgical management of ILC without compromising long-term clinical outcomes.

### Chemotherapy in ILC

In addition to surgery, systemic treatment to manage ILC is also essential. NAC is widely used in the treatment of BC in order to relieve tumor burden, allowing breast-conserving surgery to be facilitated. Specific benefits of NAC include the ability to monitor tumor response *in vivo*, predict outcome and adjust treatment regime as necessary (64). The typical outcome measurement of NAC is the achievement of pathological complete response (pCR) as this is a strong early surrogate marker for OS in BC. However, the overall consensus is that ILC responds poorly to chemotherapy with lower OS rates following NAC than observed in IDC (3, 65). This is in part due to pCR occurring less frequently in ILC subset (2). Cocquyt et al. (66) looked at the clinical response and pCR rates between IDC and ILC in 135 patients with BC. They found that the overall response for IDC was 75% compared to 50% for ILC (P = 0.0151). pCR was reported as 15% in patients with IDC and 0% in those with ILC. The type of chemotherapy used was found not to affect this. In addition, Lips et al. (67) performed an analysis of a large dataset comprising two NAC trials, 676 patients in total, of which 75 were of ILC. pCR rates were compared between IDC and ILC and showed that following NAC, ILCs were significantly less likely to achieve pCR than IDCs (11% versus 25%). The CTNeoBC meta-analysis by Cortazar et al. (68) reported that while there is a significant association between pCR and OS in BC, long-term outcomes are influenced by BC subtypes. Patients with triple-negative BC and those with HER2-positive, hormone-receptor negative tumors were found to have the strongest association between pCR and long-term outcomes. Suggesting that pCR may not be prognostic of long-term outcome in patients with ILC.

The poor chemo-sensitivity of ILC can likely be explained by its hallmark biological characteristics and the expression of specific markers. Namely, low histological grade, ER positivity, relatively low mutational burden, and a low rate of proliferation assessed with Ki-67 immunohistochemistry (5). Given the poor response rate to NAC, residual tumor volumes are a major challenge in ILC due to the greater risk of local recurrence. The inability of NAC to adequately downstage and reduce tumor burden in ILC has resulted in lower rates of breast-conserving surgery and higher rates of conversion to rescue mastectomy due to positive margins (69). If NAC is unlikely to improve rates of breast-conserving surgery in ILC then it’s use should be carefully considered given the potential life-threatening toxicities (4). Interestingly, despite responding poorly to chemotherapy, ILC patients do not have increased rates of recurrence and survival is comparable to IDC (68, 69). Many molecular tests are now available to better inform treatment decisions, which may help to avoid treating ILC patients inappropriately. An example of this is the Oncotype DX 21-gene clinical assay. A study by Thomas et al. (70) compared survival outcomes between ET and chemoendocrine therapy in patients with ILC, utilizing Oncotype Dx. The use of chemotherapy in addition to ET did not improve survival outcomes for patients with ILC compared to ET alone. Oncotype Dx testing in ILC patients also revealed most cases would receive little benefit from chemotherapy, in line with the survival outcomes observed. There remains little in the literature focusing on the role of Oncotype DX in the management of ILC, with more research this may prove a vital tool in impacting ILC treatment decisions regarding the use of chemotherapy (71).

### Endocrine Therapy in ILC

As most ILCs are strongly hormone receptor positive, treatment decisions are often in favor of endocrine-based therapy, to which a vast number of patients exhibit a good response (4, 13). Most studies have shown ET to be more beneficial when used in the adjuvant setting, particularly in post-menopausal women, due to its high correlation with reducing risk of recurrence. Yet, in the neo-adjuvant setting, ET may be warranted in order to downstage tumors, and potentially allow breast-conserving surgery (72). Classically, tamoxifen, a selective ER modulator is indicated for pre-menopausal women while an aromatase inhibitor such as letrozole or anastrozole is recommended for post-menopausal women. However, research suggests that in the case of ILC, not all endocrine therapies are of equal value. Metzger Filho et al. analyzed the effectiveness of adjuvant letrozole compared with tamoxifen in cases of patients with ILC and IDC, as part of the BIG 1-98 trial (6). Overall patients with ILC had a greater benefit from treatment with letrozole than with tamoxifen. Across the 8-year follow up period, DFS was reported at 66% for tamoxifen compared to 82% for letrozole treated ILC. While OS was 74% with tamoxifen compared to 89% with letrozole (5). These results recommend the use of an aromatase inhibitor as the treatment of choice in women with ILC and suggest the possibility of endocrine resistance with tamoxifen treatment (6). However, it is inevitable that a large number of patients will eventually become resistant to treatment regardless of the endocrine agent (73). The mechanistic drivers of endocrine resistance in ILC are as yet undefined but may be explained by tumor profiling. Tumors acquiring mutations in ESR1, ERBB2 and FGFR1 have been found to exhibit a poorer response to targeted ET (73). Sikora et al. (74) used an ER positive ILC cell line (MDA-MB-134VI) to analyze this altered response to tamoxifen. The study showed that the ER drives gene expression in ILC cells that promotes cell growth in the presence of tamoxifen. Additionally, FGFR1 signaling was vital in maintaining cell viability and therefore FGFR1 inhibition may provide a target for reversing tamoxifen resistance (5).

Endocrine treatment as a neo-adjuvant approach is less well documented however the few studies conducted suggest this may be preferable to chemotherapy (4). A retrospective study by Dixon et al. (75) of 61 ILC patients reported a 66% reduction in tumor volume following three months of neo-adjuvant letrozole and a successful breast conservation rate of 81%. This small study demonstrates the potential to increase the rate of breast conservation and in turn reduce mastectomy rates among women with ILC. ET may be further enhanced when used in conjunction with targeted therapies. Cyclin-dependent kinases (CDK) 4/6 inhibitors such as palbociclib, ribociclib and abemaciclib are currently licensed for use in advanced BCs that are ER positive and HER2 negative in combination with conventional ET. The role of CDK4/6 inhibitors in combination with ET was also explored both in the adjuvant and neoadjuvant settings. To our knowledge, four clinical trials have been published to date. NeoPalAna, a phase II trial that evaluated anastrozole monotherapy versus anastrozole plus palbociclib in ER+ HER2-ve BC patients (76), PALLET, a phase II trial which compared letrozole monotherapy versus letrozole plus palbociclib administered in different schedules (77), neoMONARCH, a phase II trial in which patients were randomized to receive either anastrozole monotherapy, abemaciclib monotherapy or their combination (78) and MONALEESA-1, phase II that compared letrozole monotherapy versus letrozole plus ribociclib in two different doses 400 and 600 mg (79). Despite the fact that none of these trials resulted in improved clinical outcomes, preliminary results have showed some analogies even though the study design, primary endpoints and number of patients differ from study to study. For example, the rate of Ki67 response and complete cell cycle arrest seemed to be higher in the combination arm compared to ET alone. Whether the better anti-proliferative effect of the CDK4/6 inhibitors plus ET translates into a better clinical outcome is still debatable.

Finally, PELOPS trial (Clinical Trial registration no. NCT02764541) is currently evaluating the use of neo-adjuvant palbociclib in combination with ET (letrozole or tamoxifen) in hormone receptor positive BC. Moreover, this trial will compare the effectiveness of letrozole versus tamoxifen in cohorts of patients with ILC, through measurement of the anti-proliferative activity. Therefore, this may prove vital in guiding and informing the future endocrine treatment of ILC.

At the last ESMO congress (2020), the results of two randomized phase III studies investigating the efficacy of CDK4/6 inhibitors in addition to standard adjuvant ET in high-risk early BC patients have been presented. In particular, MonarchE trial reported a significant advantage of 3.5% in term of invasive DFS in patients who received abemaciclib plus ET compared to those treated with ET alone (92.2% versus 88.7%) (80). Conversely, in the PALLAS trial, the addition of palbociclib to adjuvant ET did not prolong invasive DFS versus ET alone (88.2% versus 88.5%) (81). These conflicting results may be due to several differences between the two trials such as study populations (more high-risk patients enrolled in MonarchE trial), drug exposure and follow up duration. However, none of the two studies provided a subgroup analysis for patients with ILC. Key characteristics of ILC are summarized in **Table 2**.

**Table 2:** Summary of Key Characteristics of ILC.

Feature	Details
Epidemiology	Post-menopausal women Caucasian western populations have the highest rates
Incidence	Increasing incidence over past two decades 10%–15% all invasive breast cancer Most common subtype after IDC of no special type
Clinical Presentation	Often vague symptoms Ill-defined mass Tumor may be impalpable
Radiology	Mammography has a low sensitivity Ultrasound can improve detection of mammographically invisible tumors MRI valuable adjunct to assess disease extent
Treatment	Challenging to achieve surgical excision with clear margins due to the diffuse nature of the disease Generally poor response to chemotherapy Excellent response to endocrine therapy, however managing resistance is still a major challenge

## Future Directions in ILC

Improvements in understanding the distinct molecular profile of ILC are essential to facilitate the development of effective future treatments. Despite ILC responding well to ET, endocrine resistance is a challenge that must be overcome. This has led to ER transcriptional regulators such as FOXA1 and RUNX1 becoming potential targets of further research into endocrine resistance in ILC (30). In addition, the PI3K pathway may also hold the key to unlocking endocrine resistance, as mutations within this pathway occur with an increased frequency in ILC (35). In the SOLAR-1 phase III clinical trial, addition of PI3K inhibitor, alpelisib, to fulvestrant was able to prolong progression-free survival (PFS) in patients with PIK3CA-mutated, hormone receptor positive, HER2-negative BC compared to fulvestrant alone (82). Another promising area of research focusing on ILC is the use of crizotinib combined with fulvestrant. The phase II trial, known as the ROLO study (Clinical Trials registration no. NCT03620643) will determine the effectiveness of combination crizotinib and fulvestrant in shrinking E-cadherin deficient, ER-positive lobular BC and diffuse gastric cancer. Crizotinib targets alterations in the CDH1 gene that cause E-cadherin deficiency and inhibits tyrosine kinase ROS1, which is vital for cell viability, while fulvestrant blocks the ER signaling pathway on BC cells. Crizotinib is already licensed in the treatment of non-small cell lung cancer and, if found to be effective in the treatment of ILC, may result in further trials investigating the inhibition of ROS1 as a novel therapeutic strategy for ILC (83). FGFR signaling is essential to the survival of cancer cells, thus targeting aspects of this pathway may provide new therapeutic options in treating ILC. Amplifications of FGFR1 are the most common reported alterations occurring in around 14% of BCs (84, 85). Reis-Filho et al. (86) performed a comprehensive molecular analysis of 13 cases of ILC. Comparative genomic hybridization identified amplifications of 8p12-p11.2 driven by FGFR1. Using the cell line MDA-MD-134, FGFR1 expression could be inhibited through the use of small interfering RNA or a small-molecule chemical inhibitor. Overall, these data suggest that FGFR1 inhibitors may prove useful as a therapeutic in ILC. In a recent study by Hayley et al. (87) RNA sequencing of primary ILC samples revealed high expression of bromodomain protein 3 (BRD3). This was associated with poor recurrence-free survival. To further investigate this, ILC cell lines were tested with JQ1, a known potent inhibitor of bromodomain proteins. JQ1 was able to inhibit cell growth in the ILC cell lines, however some of the cells were resistant to JQ1-induced apoptosis. Following further molecular analysis, JQ1 resistant cell lines were found to express high levels of FGFR1-4. Combining JQ1 with an FGFR1 inhibitor resulted in cell death in the original JQ1 resistant cells. Therefore, the inhibition of BRD3 could represent a novel therapeutic target.

Novel therapeutic approaches such as immunotherapy are becoming more promising in the treatment of BC, particularly in triple negative cancers. However, the immune response in ILC is less well studied (88). The presence of tumor infiltrating lymphocytes has been found to correlate with a good prognosis with typically higher rates of pCR after chemotherapy. Desmedt et al. (89) recently evaluated the prevalence, levels and composition of tumor infiltrating lymphocyte and their association with clinico-pathological outcomes in ILC. The results concluded that levels of tumor infiltrating lymphocytes were significantly lower in ILC compared with IDC. Although, higher levels of lymphocytes were associated with young age, nodal involvement and high proliferation suggesting high lymphocyte infiltration is associated with a worse prognosis in ILC. This suggests that immune infiltration may play a different role in ILC compared with IDC.

Du et al. (88) recently reported that ILC exhibited a higher activity of almost all types of immune cells compared to IDC. This suggests that ILC may demonstrate a greater sensitivity to existing immune check-point inhibitors. The use of immune check-point inhibitors is the current focus of a phase II trial known as the GELATO study (Clinical Trials registration no. NCT03147040). This trial aims to assess the efficacy of combining chemotherapeutic agent carboplatin with monoclonal antibody atezolizumab in patients with metastatic ILC (90). Atezolizumab works by targeting PDL-1, an inhibitory factor that can limit the development of the T-cell response. By blocking the effects of PDL-1, atezolizumab can reduce this immunosuppressive signal and increase the body’s immune response against ILC tumor cells.

## Conclusion

ILC represents a biologically distinct subset of BC, characterized by hallmark molecular features such as loss of E-cadherin expression, ER positivity, and HER2 negativity. This unique biology directly affects initial presentation and makes early diagnosis challenging, and decisions regarding both surgical and systemic therapy are ultimately more difficult compared with other BC subtypes. Given that the biological characteristics of ILC are considered favorable, an excellent prognosis should be expected over other invasive BCs. To improve outcomes of patients with ILC more emphasis should be placed on early detection and imaging techniques to accurately evaluate disease extent given the increased propensity for multifocal and contralateral disease. Evidence highlighting the value of MRI for detection and diagnosis of ILC is emerging. More accurate imaging methods will better inform surgical decisions and may lead to an increase toward the number of breast-conversing surgeries performed and a reduction in the incidence of positive margins and subsequent rescue mastectomies. Despite these challenges, key features such as ER and PR positivity are associated with an excellent response to ET among ILC patients. Evidence suggests that the use of an aromatase inhibitor such as letrozole should be the treatment of choice in ILC due to the inevitable endocrine resistance associated with tamoxifen. In the case of advanced BC, the use of targeted therapies such as CDK4/6 inhibitors in combination with traditional ET is warranted. This is currently being investigated in the neo-adjuvant setting in the PELOPS clinical trial. It is evident that mutations within the CDH1 gene are common factors in the pathogenesis of ILC alongside mutations within the PI3K pathway. Therefore, therapies targeting these pathways represent an attractive option in ILC patients. Furthering our understanding of the unique biology of ILC is essential to facilitate the development of novel therapeutic strategies, moving toward precision medicine for patients diagnosed with all subtypes of ILC.

## Author Contributions

NW: conceptualization, interpretation of data, and writing original draft. AI and AD: review, interpretation of data, and editing. OO: conceptualization, writing original draft, review, editing, and supervision. All authors contributed to the article and approved the submitted version.

## Conflict of Interest

The authors declare that the research was conducted in the absence of any commercial or financial relationships that could be construed as a potential conflict of interest.
